# Sealing Ability of Endodontic Cements: An In Vitro Study

**DOI:** 10.1155/2020/5862598

**Published:** 2020-02-13

**Authors:** Amira Kikly, Sabra Jaâfoura, Dorra Kammoun, Saida Sahtout

**Affiliations:** ^1^Department of Conservative Odontology, ABCDF Laboratory, Faculty of Dental Medicine, University of Monastir, Monastir 5000, Tunisia; ^2^Department of Dental Biomaterials, ABCDF Laboratory, Faculty of Dental Medicine, University of Monastir, Monastir 5000, Tunisia

## Abstract

The root canal system must be obturated using a hermetic seal to prevent the penetration of microorganisms and bacterial toxins into the endodontic system. The principles of adhesive dentistry have been increasingly used in endodontics. In fact, resin-based sealers are increasingly used. The objective of this study was to evaluate, in vitro, the sealing ability of resin cement in comparison with calcium hydroxide-based cement. *Materials and Methods*. Eighty root canals were prepared with the Tilos system and were randomly divided into four groups according to the filling material. The best combination was evaluated on the basis of its sealing ability. The dye infiltration degree was evaluated using both a stereomicroscope after diaphanization and the dye rise test. *Results*. A significant difference was observed between the four obturation systems with regard to the number of infiltrated walls (*p*=0.014) and the infiltration depth (*p*=0.025). The group of teeth obturated with EndoREZ® and EndoREZ® gutta cones differ significantly from the group obturated with EndoREZ® cement and gutta-percha cones in terms of apical sealing (*p*=0.011). A significant difference was also observed between the group of teeth obturated using EndoREZ® gutta cones and EndoREZ® cement and the group of teeth obturated with EndoREZ® cement (*p*=0.026). *Conclusion*. When used with EndoREZ® gutta cones, EndoREZ® cement showed the best sealing ability, particularly in the apical region. When used with gutta-percha cones, Acroseal and EndoREZ® cements exhibited similar sealing abilities.

## 1. Introduction

The success of any endodontic therapy certainly depends on the root canal preparation. However, there is a close relationship between the root canal preparation and obturation, as three-dimensional hermetic filling is related to the root canal trimming and shaping [1].

The root canal system must be obturated using a hermetic seal to prevent the penetration of microorganisms and bacterial toxins into the endodontic system [2]. Pastes associated with gutta cones are best indicated for this type of obturation. These materials must be biocompatible and nonresorbable, so that they can act as a dressing in the transition zone between the obturation and the periapical tissue [3, 4]. The best-known and most commonly used filling material is gutta-percha. However, it cannot hermetically seal the root canal. Regardless of the root canal filling technique used, insufficient hermetic areas are observed if no sealer is used. Consequently, a root canal filling must essentially consist of a basic material in the form of one or more cones and a root canal filling paste. The function of the latter is to fill the vacuities between the root wall and the cone while maintaining a good dimensional stability [3, 5, 6]. At least 59% of endodontic failures can be attributed to apical percolation induced by leakage. The penetration of microorganisms and their toxins at the level of the obturated root canal is manifested by the presence of bacterial grounds escaping the body's defense system [7].

The principles of adhesive dentistry have been increasingly implemented in endodontics. In fact, micromechanical retention allows to improve the long-term root canal sealing and thus avoids any bacterial recontamination [8, 9].

Currently, resin-based sealers are increasingly used. The concept of creating a monoblock in the root canal aims at having a perfect sealing of the intracanal space and at avoiding the lack of chemical union between the gutta-percha polyisoprene and the methacrylate resin-based sealer. A stress analysis and mechanical evaluation of endodontically treated teeth revealed that the stress was concentrated where the load was applied to the tooth, at the interface between the crown and the dental root and in the upper zone of the endodontic material. The main differences and the higher stresses were found in the endodontic material-cement interface [10]. The study by Chieruzzi et al. confirmed that the strength of the dental systems subjected to masticatory loads was strictly related to the bond at the interface post/cement and cement/dentin [11].

Gutta-percha cones coated with an adhesive consisting of methyl polybutadiene diisocyanate and a hydrophilic adhesive resin have been introduced in the market. Thus, a strong chemical union is achieved between the gutta-percha and the methacrylate resin-based sealer which allows the formation of a solid monoblock. This concept is found in the EndoREZ® system.

One of the most widely used methods for assessing the ability of these materials to provide a good sealing in vitro is the study of apical infiltration during dye penetration. It is a linear measurement of dye penetration between the root canal walls and the root canal filling material. The dye that is most used is the Indian ink, thanks to its weak molecular weight [12].

The objective of this work was to evaluate in vitro the resin cement sealing (EndoREZ® (Ultradent, United States)) in comparison with calcium hydroxide-based cement (Acroseal (Septodont, France)). The first null hypothesis was that EndoREZ® and Acroseal® are equal in sealing ability. The second was that EndoREZ® has the same sealing ability when used with EndoREZ® gutta-percha cones or gutta-percha cones.

## 2. Materials and Methods

Eighty maxillary and mandibular mono- and biradicular teeth that were caries free and freshly extracted were chosen according to the radiological criterion (absence of pulpal calcification). These teeth were stored in potassium hypochlorite at 0.9% until the beginning of the operative procedure. They were randomly divided into four groups. The length of each tooth was determined with precision using a vernier caliper. After preparing the access cavities, the working length was accurately determined on all the root canals using a *n*° 8-*k* type file which was introduced at each canal until passing the apical foramen, then it was slightly removed up to this level. Later, this length was measured by an endodontic ruler. The root canals were prepared using the Tilos® system, a hybrid system combing stainless steel files and nickel titanium files. A counter angle at 30° reciprocity was used for shaping.

The preparation protocol recommended by the manufacturer is as follows:

- Initial preparation was done using *k* type files *n*°10, *n*°15, *n*°20,
- Measurement of the apical foramina diameters was done by apical files,
- A hand *n*°15-*k* type file was used, shaping S2 files and S3 files,
- A hand *n*° 20-*k* type file was used, then irrigated with sodium hypochlorite, and then the *n*° 10-*k* type file was withdrawed,
- The working length was determined by using a transitional file *n*° 25/8% and a transitional file *n*° 25/4% on the working length. Shaping was finished using apical files.

During root canal shaping, the tip of each instrument was coated with ethylenediaminetetraacetic acid (EDTA). 2.5% sodium hypochlorite was also used before and after each instrumental penetration. At the end of the root canal shaping, just before beginning root canal filling, irrigation with EDTA at 17% for 2 min was carried out. It was followed by rinsing with distilled water, irrigation with sodium hypochlorite, final rinsing with distilled water, and eventually drying. Later, the teeth of the four groups were obturated as follows:

- Group 1: the root canals were obturated using EndoREZ® cement and resin-coated EndoREZ points (EndoREZ points are standard ISO-sized gutta-percha points coated with a thin resin coating, which bonds chemically to the EndoREZ canal sealer).
- Group 2: the root canals were obturated using EndoREZ® cement and nonstandardized gutta-percha.
- Group 3: the root canals were obturated using Acroseal cement and nonstandardized gutta-percha points.
- Group 4: the canals were obturated using only EndoREZ® cement.

Coronal obturation was performed using a composite resin (Herculite™ classic microhybrid composite: Kerr) to minimize as much as possible the dye microinfiltration through the access cavity. Retro-alveolar radiographs were taken to evaluate the root canal and coronal obturation according to the ray perpendicularity rule with regard to the radiographic film.

Later, all the teeth were immersed in Chinese ink “le coq®.” Each tooth was placed, with the apex directed upwards, in a Pyrex test tube (12 mL) that was half filled with Indian ink. Then, each test tube was placed in a GFL® 3019 shaker for 10 min. After immersion, the teeth were dried in the air for 24 hours. The dye deposits on the root surface of each tooth were carefully removed using finishing discs. All the teeth underwent sectioning of their crowns using diamond discs mounted on a counter angle. This was performed in order to not to exhaust the acid solution.

Each group of samples was placed for 30 days in nitric acid at 5% under continuous shaking and at room temperature. The acid solution was renewed daily. To control the complete decalcification of the roots, a *n*° 6-*k* type file was introduced into a control root. It should get into the dentin without resistance, as it would get into butter. After this procedure, the teeth were thoroughly rinsed with running water for 2 h. A progressive and complete dehydration of the roots was carried out using ethanol solutions in an increasing way (75%, 85%, 95%, and pure ethanol) for 24 hours each.

**Figure 1:**
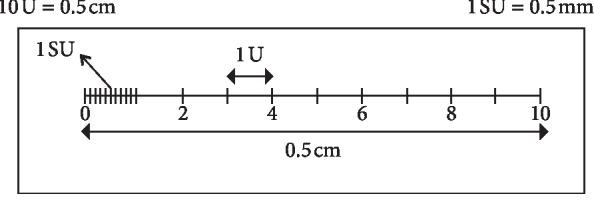
Micrometric scale.

Finally, each group of teeth was placed in a Petri dish containing a methyl salicylate solution for 24 hours to make the tooth root transparent. Each tooth in each group was placed in a Petri dish containing a methyl salicylate solution, and it was placed under the stereomicroscope focus (Zeiss Stemi 2000-c, ZEISS, Jena, Germany) to be examined and to evaluate the dye infiltration degree. The different teeth in each group were photographed at the stereomicroscope by an adaptable photographic system. The dye rise measures were taken under a 2, 1, or 0.8x magnification per unit of the micrometric scale. They were then converted into millimeters. Under 2x magnification, the measures were converted according to the data summarized in Figure 1.

Statistical data were performed using the data processing software: SPSS statistics 17.0 (SPSS Statistics for Windows, Version 17.0. Chicago: SPSS Inc.). Microsoft Office Excel 2007 software was equally used to establish some numerical functions and descriptive graphic representations.

## 3. Results

**Figure 2:**
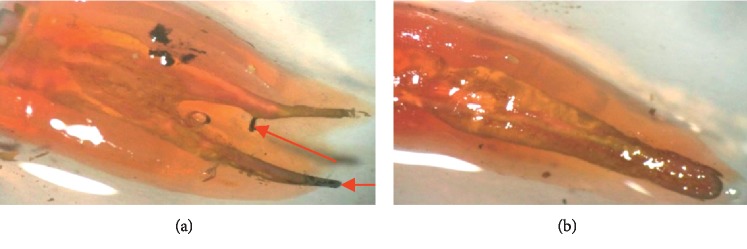
Examples of stereomicroscopic observation of teeth in group 1. (a) Complete and homogeneous obturation and dye infiltration at the apical level and at the lateral root canal. (b) Complete and homogeneous obturation and the cement covers all the intraradicular walls as well as the EndoREZ® gutta cones. No dye infiltration.

**Figure 3:**
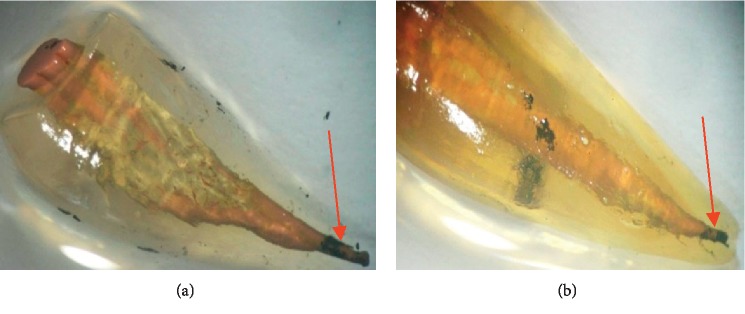
Examples of stereomicroscopic observation of teeth in group 2. (a) An important layer of cement covering the intracanal walls with a stack of GP cones. Ink infiltration does not exceed 1 mm. (b) A slight infiltration at the apical level, insufficient external cleaning, and complete homogeneous obturation.

**Figure 4:**
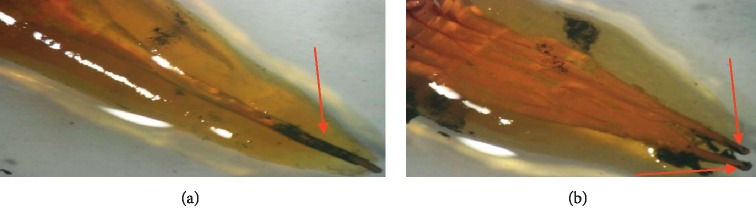
Examples of stereomicroscopic observation of teeth in group 3. (a) Insufficient external cleaning, apical infiltration, and complete homogeneous obturation. (b) Insufficient external cleaning, gutta-percha cones are piled one over the other, complete homogeneous obturation, and slight apical infiltration.

**Figure 5:**
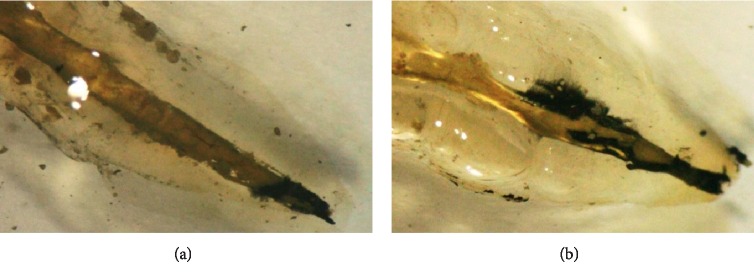
Examples of stereomicroscopic observation of teeth in group 4. (a) Inhomogeneous and complete obturation with slight infiltration at the apical level. (b) Significant dye infiltration on two thirds of the root canal.

The results of the stereomicroscopic observation of the different groups are presented in Figures 2–5.

**Figure 6:**
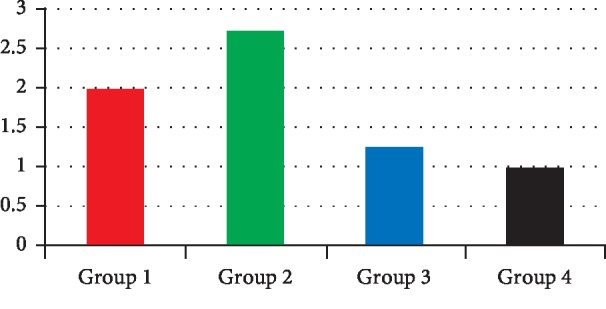
Histogram showing the variation in the infiltration depth according to the group of teeth.

**Figure 7:**
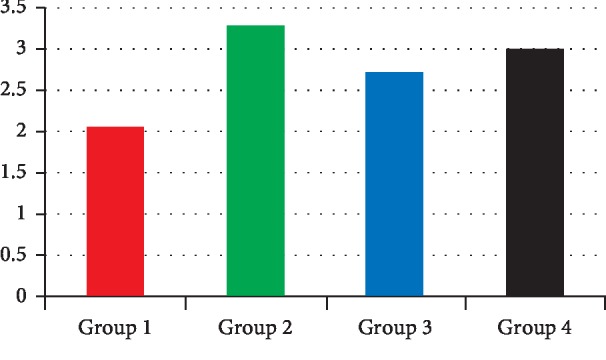
Histogram showing the variation in the number of infiltrated walls according to the group of teeth.

The average depth of infiltration was 3.16 mm with an interval ranging from 0 mm to 12.5 mm. The average number of infiltrated walls was 3.03 with a minimum of 4 walls. The EndoREZ® gutta-percha group had the most significant average of infiltration value (2.73) (Figure 6). As for the average number of infiltrated walls, the EndoREZ® gutta-percha group presented the highest average (3.3) (Figure 7). The one-factor ANOVA showed a significant difference between the 4 obturation systems regarding the number of infiltrated walls (*p*=0.014) and the infiltration depth (*p*=0.025).

**Figure 8:**
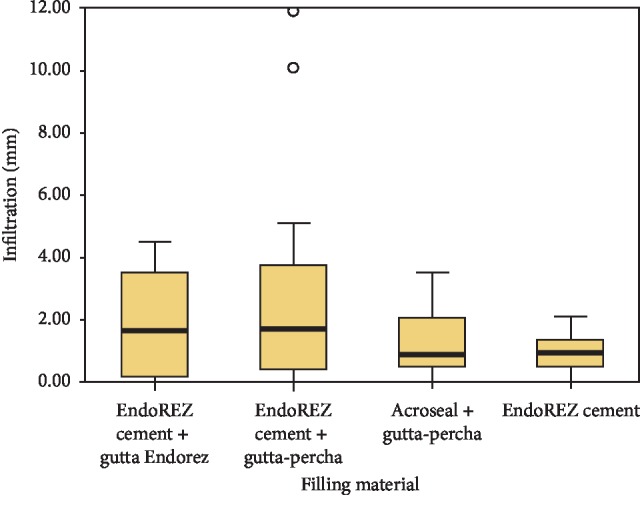
A box plot showing the distribution of the infiltrated values around the average of each obturation system, as well as the maximum and minimum values.

The box plot (Figure 8) shows the distribution of the infiltration values around the average for each obturation system. The comparison of averages between the different groups of teeth, two by two, showed significant variations for the following combinations:

- EndoREZ® + G. EndoREZ®/EndoREZ® + G. percha (*p*=0.011)
- EndoREZ® + G. percha/EndoREZ® (*p*=0.026)

## 4. Discussion

The null hypotheses were rejected. There were statistical differences in the infiltration depth and the number of infiltrated walls between the different combinations: EndoREZ®, Acroseal®, and EndoREZ® gutta-percha cones or gutta-percha cones.

It has been reported that the prevalence of healing after initial treatment and retreatment of root canals is 86% and 82%, respectively [13]. Causes of endodontic failure can be classified into biological and technical factors. Failures related to microorganisms can be caused by anatomical difficulties such as isthmus, apical ramification, and other morphological irregularities [14]. The main objectives of endodontic treatment are to maintain or recover the integrity and health of teeth and supporting tissues through the reduction or elimination of microorganisms from the root canals and prevent reinfection (AAPD 2016/2017). Thus, sealing ability of root canal filling is important [15].

Sealing was assessed through the capillarity phenomenon. It is the linear measurement of the Chinese ink penetration at the interface root canal walls-material [16].

The clarification of teeth is an easy-to-use and faithful method compared to other techniques using longitudinal or transversal sections because the root canal obturation for this technique is examined on all the sides and the detection of accessory canals or cracks is easy to be seen [17]. During the elimination of the Chinese ink deposits, it was difficult to clean the outer surface of multiradicular teeth, mainly at the furcation level. At the stereomicroscope, this coloration gives the illusion of an infiltration, which can distort the measures. To avoid any possible confusion, it was necessary to apply two varnish coats on the root surface, with the exception of the last millimeters, before immersion in Indian ink. The choice of the lateral condensation technique can be considered as the best compromise in the daily practice of a general practitioner. It has been shown that the forces exerted during lateral compaction do not practically affect the sealing of the root canal obturation [18].

Sealing is better in the coronal part than in the apical part. This can be explained by two reasons. Firstly, the dentinal tubules density is significantly higher in the coronal part than in the middle and apical part. Secondly, the smear layer is more easily removed in the coronal area [9]. The use of the sealer is conditioned by the good mixing of the appropriate volumetric catalyst/base ratio (for Acroseal cement (Septodont, France)) and by the use of Skini Syringe and the good choice of the length as well as the needle diameter (for EndoREZ® cement).

The use of the EndoREZ® accelerator could have negative effects on its sealing [19]. Similar to all light-curing materials, polymerization shrinkage is a factor that reduces adhesion between the parietal dentin and the sealer [8]. To minimize as much as possible the disadvantages of the catalyst, taking into consideration the hydrophilic nature of EndoREZ®, it is recommended to maintain a degree of humidity at the dentin level, thus allowing the cement to penetrate deeply into the dentinal tubules. In their study, Gillepsie and Loushine showed that the addition of dual self-etching to the EndoREZ® system improves its sealing by avoiding the formation of hiatus resulting from the polymerization shrinkage [20].

When using EndoREZ® cement and gutta-percha for root canal obturation in group 2, the average infiltration depth reached 2.73 mm, which is significantly higher (*p*=0.026) than that found in group 1 (average 1.97). According to Mutal and Gani, the interface between the resin-coated gutta-percha cones (EndoREZ® gutta) and the resin sealer represents a weak point manifested by the appearance of voids in the form of microvacuoles during the polymerization shrinkage of the cement, resulting in a lack of sealing [21]. It is recommended to use EndoREZ® cement with the corresponding cones due to the lack of chemical union between the gutta-percha polyisoprene and the methacrylic resin-based sealers.

EndoREZ® gutta cones are coated with a polybutadiene diisocyanate-methacrylate adhesive. This adhesive contains a hydrophobic portion, chemically compatible with gutta-percha polyisoprene and another hydrophilic portion, chemically compatible with the hydrophilic character of methacrylic resin-based sealers [22, 23]. EndoREZ® is a dual polymerization hydrophilic sealer containing zinc oxide, barium sulfate, resins, and pigments in a urethane dimethacrylate resin matrix. It can be used in the humid environment of the root canal system, and it is effective in penetrating the dentinal tubules and in closely fitting the canal walls [24].

Restrepo-Restrepo et al. reported that irrigation with 2.5% sodium hypochlorite results in a decrease in the microhardness of the dentin. This is due to the ability of this irrigation agent to dissolve the organic component of the dentin, in particular, the collagen which adversely affects the quality of the sealer's adhesion and contributes to the decrease of the micromechanical interaction between the resin sealing cement and the dentin [4]. The humid state of the root canal at the obturation time has a very significant effect on the microleakage of the root canals filled with resin-coated gutta-percha/EndoREZ®, leading to a significant increase in the sealing performance [18, 25]. Root canal system obturation by a material having adapted physical and biological properties is the main goal of any endodontic treatment. In fact, root canal obturation using EndoREZ® and EndoREZ® gutta cones has given infiltration values comparable to those obtained with Acroseal and gutta-percha cones.

The difference between group 1 and group 4 was not significant (*p*=0.062). When using EndoREZ® or Acroseal® in combination with gutta-percha cones, apical sealing was comparable. This finding is in accordance with the one found in the study by Scarparo et al. [26]. The sealing of methacrylate resin-based root canal cement provides a better resistance to bacterial percolation than calcium hydroxide-based cement [12, 27]. The latter does not completely prevent the percolation of periapical fluids, which could be responsible for the porosities and voids within the obturation [27].

The presence of voids on the cement surface is more frequent with calcium hydroxide sealers [28]. According to Eldeniz and Ørstavik, the ability to ensure a tight seal between Apexit® cement (Ivoclar Vivadent), which is calcium hydroxide-based cement, and the dental walls is better when compared to EndoREZ® cement [29]. However, according to the study conducted by Pinna et al., Epiphany™ and EndoREZ® cements provide better sealing qualities than Acroseal cement [9].

With regard to the clinical performances, a retrospective and radiographic study suggests that EndoREZ® used in conjunction with gutta-percha cones presents similar performances to those of conventional endodontic sealants for up to 8 years [30].

The manufacturer recommended that the root canal walls be kept moist, not dehydrated, to take maximum advantage of the hydrophilic properties of the EndoREZ, thus allowing for resin sealer tag penetration. In this study, the canals were dried with paper points till it came out dry. We did not control the moisture degree of root canal walls. Future studies are indicated to investigate the long-term sealing ability of EndoREZ. Future research may have to focus on the modifications that are liable to achieve a better sealing ability imparted to endodontic filling materials, specifically those which target the accomplishment of a true monoblock system.

## 5. Conclusions

When used with EndoREZ® gutta cones, EndoREZ® cement showed the best sealing ability, especially in the apical region. When used with gutta-percha cones, Acroseal and EndoREZ® cements exhibited similar sealing abilities.

## Data Availability

The experimental data used to support the findings of this study are included within the article.
